# Data profile: the Korean Workers’ Compensation-National Health Insurance Service (KoWorC-NHIS) cohort

**DOI:** 10.4178/epih.e2024071

**Published:** 2024-08-19

**Authors:** Jeehee Min, Eun Mi Kim, Jaiyong Kim, Jungwon Jang, Youngjin Choi, Inah Kim

**Affiliations:** 1 Department of Occupational and Environmental Medicine, Hanyang University Hospital, Seoul, Korea; 2 Department of Big Data Management, National Health Insurance Service, Wonju, Korea; 3 Institute for Health and Society, Hanyang University, Seoul, Korea; 4 Department of Public Health Sciences, Hanyang University Graduate School, Seoul, Korea; 5 Department of Occupational and Environmental Medicine, Hanyang University College of Medicine, Seoul, Korea

**Keywords:** Cohort studies, National Health Insurance Database, Occupational injuries, Accident, Occupational, Precarious employment

## Abstract

The Korean Workers’ Compensation-National Health Insurance Service (KoWorC-NHIS) cohort was established to investigate the longitudinal health outcomes of Korean workers who have been compensated for occupational injuries or diseases. This cohort study, which utilized data spanning from 2004 to 2015, merged workers’ compensation insurance claim data with the National Health Insurance Database (NHID), encompassing 858,793 participants. The data included socio-demographic factors such as age, sex, income, address, insurance type, and disability grade. It also covered the types of occupational accidents, International Classification of Diseases, 10th revision codes for diseases or accidents, work tenure, industry, occupation code, and company size. Additional details such as the occupational hire date, date of claim, date of recognition, and affected body parts were recorded. The cohort predominantly consisted of male workers (80.0%), with the majority experiencing their first occupational accident in their 40s (27.6%) or 50s (25.3%). Notably, 93.1% of the cases were classified as occupational injuries. By integrating this data with that from the NHID, updates on health utilization, employment status, and income changes were made annually. The follow-up period for this study is set to conclude in 2045.

## INTRODUCTION

Industrial accidents have declined but remain disproportionately prevalent among vulnerable, low-status workers due to labor market polarization, which frequently involves irregular or short-term employment through subcontractors [1-5]. Outsourcing complicates the legal responsibility for worker safety and disrupts safety management systems, potentially leading to regulatory failures [6-10]. Precarious employment complicates the tracking and assessment of long-term occupational health effects due to frequent job changes and the difficulty in identifying employers [10].

Using data from the National Health Insurance Database (NHID), we constructed a cohort to explore the health effects of occupational characteristics on precarious workers in Korea. The country’s single-payer social insurance system, which encompasses both workers’ compensation insurance and national health insurance, facilitates detailed tracking of workers across various employment statuses, including day laborers and part-time workers [11,12]. Despite the extensive coverage of 19.87 million wage workers, representing 67.7% of the workforce, challenges remain in covering the entire economically active population [13,14]. We conducted a longitudinal assessment of healthcare behaviors and disease incidence by linking health utilization data with workers’ compensation insurance records. This dataset includes all Korean industrial accident workers and offers unique insights into changes in income, occupational transitions, health behaviors, and workplace risks. Our research aimed to elucidate both the immediate and long-term health impacts of industrial accidents, particularly for precarious workers, who are often overlooked in traditional studies.

## DATA RESOURCE AND POPULATION COVERAGE

### Data collected

Workers’ compensation insurance claim data were merged with the NHID using resident registration numbers in this cohort. The merging was conducted by the Expert Data Combination Agency in accordance with the Enforcement Decree of the Personal Information Protection Act.

The Korea Workers’ Compensation and Welfare Service (COMWEL) administers workers’ compensation under the Industrial Accident Compensation Insurance Act. This act provides coverage for medical expenses and survivor compensation in cases of occupational injuries and diseases. The COMWEL assesses claims by examining employment records, as well as statements from workers and their coworkers, and conducts site inspections when necessary. Decisions on industrial accidents are determined according to established guidelines or, in more complex situations, by a committee of experts.

All information was collected from the compensation data when occupational injuries occurred. This cohort study included workers who received compensation from COMWEL during the period from 2004 to 2015.

To track health outcomes, we combined approved occupational accident (occupational injury and occupational disease) compensation data with figures from the NHID. We linked the NHID and compensation databases using personal IDs. The data merging was conducted by a specialized agency, the National Health Insurance Service (NHIS), in accordance with the Enforcement Decree of the Personal Information Protection Act. All data were securely encrypted and processed. Korea operates a universal health insurance coverage system, under which the entire population is covered by the National Health Insurance Act [12]. In this mandatory system, all medical institutions in Korea are required to have contracts with the government. Medical services are paid through a regulated fee-for-service system, and insurance premiums are determined based on income levels. A population-based database from the NHIS was compiled using the NHID. It includes sociodemographic characteristics, medical treatment records, and income proportions of the population. Since the data were not updated in real time, the researchers selected the data period based on the analysis timing. For this study, data were included through December 31, 2022. The follow-up period is subject to updates.

**Figure 1.**
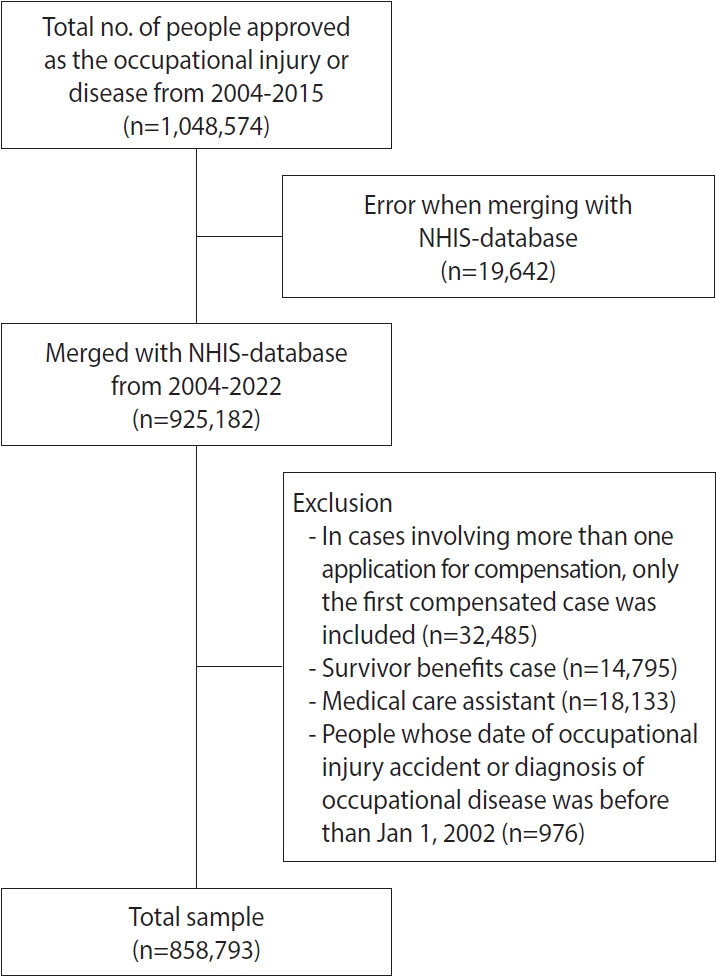
Korean Workers’ Compensation-National Health Insurance Service (KoWorC-NHIS) flow chart. NHIS, National Health Insurance Service.

In this cohort, workers who received approval for workers’ compensation were included. We excluded cases involving survivor benefits. If a worker had applied for compensation more than twice, only the first approved case was considered for inclusion. Among the total population, 781 individuals were approved for workers’ compensation twice, 15 were approved 3 times, and 2 were approved 4 times. Additionally, individuals who received medical assistance were not included in the study. We also excluded workers whose accident dates occurred before 2002, as the NHIS-claimed data prior to this year were not formatted in a way that was suitable for our research (Figure 1).

### Variables

The Korean Workers’ Compensation-National Health Insurance Service (KoWorC-NHIS) cohort study incorporated sociodemographic variables such as sex, age, insurance type, income, and region of residence and socioeconomic indicators from the NHID. This setup facilitated longitudinal analyses (Table 1).

We extracted the database from the NHID, spanning from the cohort entry point to the censoring point, using personal ID as the merging key. The cohort database comprises 6 tables: eligibility, cause of death, medical treatment, health examination, medical care institution, and occupational accidents. The eligibility table contains data on sex, age, residence, income, insurance type, and disability status. The cause of death table records the cause and date of death. The medical treatment tables catalog diagnosis codes for diagnosed conditions, classifications for inpatient, outpatient, and emergency room visits, treatments and medications administered during those encounters, and associated medical costs. The health examination table includes information on past medical and family history, health behaviors such as smoking, drinking, and physical activity, as well as health examination measurements like height, weight, body mass index, chest X-rays, blood pressure, fasting blood sugar, and other laboratory test results. The medical care institution table includes information about the type of institution, location, number of beds, facilities, and number of physicians.

Data in the occupational accidents table are sourced from the COMWEL compensation database. This table details occupational injuries or diseases, with variables fixed at the time of the incident. Consequently, each ID is assigned a single value that reflects the circumstances at the time of the industrial accident. Occupational data and company information, including details such as International Classification of Diseases, 10th revision codes, occupation codes, tenure, accident/diagnosis dates, and duration of compensation claims, were sourced from the Workers’ Compensation Database. Occupation and industry classifications were manually converted to facilitate international comparisons.

To adjust for comorbidities that significantly affect disease prognosis and act as confounders or mediators in the studies, both disability severity and the Charlson comorbidity index score were utilized. Comorbidity data, obtained from national insurance records, were timed variably based on the specifics of each study. Impairment severity was assessed post-treatment by COMWEL physicians, who evaluated the compensation levels.

### Entry and censoring points

The cohort entry date was defined as either the date of the occupational injury or the date of occupational disease diagnosis, as recorded in the COMWEL database. The censoring point was set at the earlier of 2 dates: the date of death or December 31, 2022. All participants were followed from their entry date to the censoring point. Person-years were calculated using the follow-up period (Table 2). The total person-years amounted to 13,223,234. Additionally, the average follow-up duration was 13.5 years.

### Ethics statement

This study involving human participants was reviewed and approved by the Institutional Review Board of Hanyang University, Korea (study No. HYUIRB-202012-005-4).

## DATA RESOURCE USE

The baseline characteristics of the study participants are detailed in Table 3. The study included a total of 858,793 participants, with males comprising 80.0% of the sample. The age distribution showed that most participants experienced their first occupational accident either in their 30s (n= 180,490, 21.0%) or their 50s (n= 216,928, 25.3%). The majority were paid workers (n = 641,730, 74.7%), while self-employed workers accounted for 25.3% of the sample (n= 217,063). Regarding income levels, 24.3% of the participants (n= 205,634) were in the lowest 20%. Geographically, 38.5% of the participants resided in the Seoul metropolitan area (n=329,027), 30.4% lived in other metropolitan areas (n= 260,174), and the remaining 31.1% lived in non-metropolitan cities (n= 265,759).

Regarding the characteristics of occupational accidents, the majority were occupational injuries (n= 799,521, 93.1%), as opposed to occupational diseases (n= 59,272, 6.9%). The Korea Workers’ Compensation and Welfare Service approved 98.4% of all reported occupational injuries or diseases within 3 months of the application date. In the total cohort of participants, trauma (S00-T98, V01-V98) was the most common (92.1%).

As relates to job characteristics, 35.4% of the participants were employed in the manufacturing industry. Elementary workers (n= 329,855, 38.4%), constituted the largest group in the occupational accident cohort. The majority of these workers had been employed for less than 5 years (n= 730,508, 85.1%). Most participants in this cohort experienced work-related injuries. The pre-trained workforce was particularly vulnerable to occupational accidents [15].

Regarding comorbidities and the severity of disability, 60.7% of the study population had no disabilities. Additionally, 68.6% had no other diagnosed diseases at the time of the occupational accidents (Table 3). There were 10,387 deaths, representing 1.2% of the 858,793 individuals in the cohort.

## STRENGTHS AND LIMITATIONS

Occupational accidents tend to occur among relatively vulnerable workers. This group can be monitored to observe long-term health outcomes and socioeconomic changes, such as variations in income, residence, and employment status, particularly among workers with lower levels of employment who have experienced occupational accidents. Numerous studies have indicated that precarious employment, which includes contingent, indirectly employed, and subcontractor workers, carries a greater risk of exposure to industrial accidents or diseases compared to direct employment [2,5,16].

Despite the significance of precarious employment, constructing a long-term follow-up cohort study for workers in such positions presents considerable challenges. It is particularly difficult for researchers to track variables like employment status, income, and participation in long-term surveys. Consequently, this cohort, which integrates 2 social insurance systems, offers valuable insights into the health impacts and rates of occupational accidents through its ability to conduct long-term follow-up with large study populations.

Due to employment insecurity, atypical workers often face excessive workloads and understaffing compared to their regular counterparts. Additionally, many hazardous tasks that regular workers avoid are frequently outsourced to small companies [5,17]. The unclear nature of the employment relationship results in both parties shirking responsibilities related to safety control and management [17]. In addition, workers in the early stages of labor market entry are often unskilled [2]. For these reasons, numerous studies have highlighted a strong correlation between atypical labor and occupational accidents [2,5].

This study allows the examination of various topics due to the availability of information about the workplaces where workers were employed at the time of their occupational injuries. It is feasible to assess specific industries for occupational cancers, acute poisonings, and respiratory diseases, among others. Cohorts can be utilized to evaluate both short-term and long-term health effects over periods of 10 years or more, taking into account the time it takes for exposure to an occupational hazard to result in an occupational disease. Moreover, occupational injuries have a significant impact on workers’ lives. It is possible to examine whether these injuries lead to changes in employment status, employment levels, income, and residence, including job losses. For instance, researchers can investigate the risk factors that influence worker unemployment and changes in employment levels following an industrial accident. If the goal is to identify the risk of cancer incidence associated with high chemical exposure, researchers can link these data to occupational and industry-specific exposure data, such as the Korean Carcinogen Exposure (CAREX) dataset [18].

This cohort reflects comprehensive data on workers who have received compensation. The baseline data were collected from all compensated workers between 2004 and 2015. Moreover, this is the inaugural cohort study in Korea dedicated to monitoring the long-term health outcomes of workers who have suffered occupational injuries or diseases. By integrating this cohort with NHIS data, researchers can evaluate the incidence of specific diseases, such as cancer or psychiatric disorders, which are challenging to assess over short-term follow-up periods. The integration with NHIS data, consisting of administratively collected insurance claims, ensures that the data are not subject to recall bias, a significant limitation often encountered in survey-based studies.

However, the current dataset lacks a control group, which complicates the determination of causality. Depending on the specific hazard, it may be feasible to define a non-exposed group within the cohort for manipulative purposes. Given the substantial presence of service industry workers in the cohort, they could potentially serve as a control group. This arrangement would enable comparisons across different occupations, such as manufacturing or construction, particularly concerning physical and chemical hazards. However, since they do not represent the entire workforce, the findings are only relevant to this specific context and cannot be broadly generalized.

In addition, because of the systematic procedures of worker compensation, selection bias may have occurred as minor occupational accidents or diseases might have been overlooked. Under the Industrial Accident Compensation Insurance Act, only work-related injuries or diseases necessitating more than 3 days of hospitalization were eligible for compensation [11]. For this reason, as a previous study pointed out, less severe occupational injuries or diseases such as contact dermatitis, toxic intoxication manifesting merely as dizziness, and corneal damage from chemical spills are under-reported in the compensation database. Thus, these less severe cases were excluded from the cohort. This selection bias should be carefully considered when interpreting our results. In this study, control groups were not selected. Future research utilizing this cohort could address this limitation by selecting control groups from the NHID using various matching methods.

Despite these limitations, a cohort that integrates data from Korea’s workers’ compensation insurance system with data from the NHIS can be utilized to more closely assess the health effects and changes in the socioeconomic status of vulnerable labor groups.

This cohort will also allow us to better assess the health impacts and socioeconomic statuses of vulnerable labor groups.

## Figures and Tables

**Table 1.** Variables for the Korean Workers’ Compensation-National Health Insurance Service (KoWorC-NHIS) cohort

Database	Category	Variable
Eligibility	Demographic characteristics	Age
		Sex
		Insurance type
		Income
		Residence
		Disability
Death	Death	Date of death
		Cause of death
Healthcare	Healthcare access	Disease (ICD-10)
		Inpatient/Outpatient/Emergency
		Treatments and medications
		Medical expenses
Medical examination	Medical history and health behavior	Medical history
		Family history
		Smoking
		Alcohol consumption
		Physical activity
	Physical measurements and laboratory exam	Height/Weight/BMI
		Chest X-ray
		Blood pressure/Fasting blood sugar
		Other laboratory exam
Healthcare center	Category of healthcare center	-
	Size of the healthcare center	-
Occupational accident	Characteristics of occupational accident	Occupational injury or disease
		Claims processing time
		ICD-10 for occupational injury or disease
	Characteristics of jobs	Working year
		Industry
		Occupation
		Company size
	Characteristics of comorbidity and severity of impairment	Charlson comorbidity index score
		Severity of impairment

ICD-10, International Classification of Diseases, 10th revision; BMI, body mass index.

**Table 2.** Population and person-years (PY) divided by the enrollment year

Enrollment year	Total population (n)	Sum of PY	Average of PY
2004	80,484	1,522,031	18.9±0.8
2005	77,536	1,389,815	17.9±0.7
2006	80,382	1,361,232	16.9±0.7
2007	80,675	1,286,006	15.9±0.6
2008	86,125	1,287,865	15.0±0.6
2009	87,943	1,226,885	14.0±0.5
2010	88,934	1,153,106	13.0±0.4
2011	84,510	1,011,880	12.0±0.4
2012	83,848	920,549	11.0±0.3
2013	84,726	845,793	10.0±0.3
2014	83,533	750,461	9.0±0.2
2015	58,542	467,611	8.0±0.2

**Table 3.** Characteristics of the study population

Characteristics	Total	Male	Female
Total	858,793 (100)	686,958 (80.0)	171,835 (20.0)
Age at occupational accident (yr)			
Mean±SD	45.2±12.5	44.4±12.4	48.2±12.5
10s	11,739 (1.4)	10,525 (1.5)	1,214 (0.7)
20s	108,093 (12.6)	89,415 (13.0)	18,678 (10.9)
30s	180,490 (21.0)	159,764 (23.3)	20,726 (12.1)
40s	237,129 (27.6)	189,052 (27.5)	48,077 (28.0)
50s	216,928 (25.3)	161,993 (23.6)	54,935 (32.0)
60s	89,874 (10.5)	66,565 (9.7)	23,309 (13.6)
≥70s	14,540 (1.7)	9,644 (1.4)	4,896 (2.8)
Insurance type			
Paid worker	641,730 (74.7)	494,609 (72.0)	147,121 (85.6)
Self-employed	217,063 (25.3)	192,349 (28.0)	24,714 (14.4)
Income (%)			
Upper 80	640,390 (75.7)	539,128 (79.7)	101,262 (59.8)
Under 20	205,634 (24.3)	137,517 (20.3)	68,117 (40.2)
Address			
Seoul metropolitan	329,027 (38.5)	257,609 (37.7)	71,418 (41.7)
Other metropolitan	260,174 (30.4)	210,879 (30.8)	49,295 (28.8)
Non-metropolitan city	265,759 (31.1)	215,170 (31.5)	50,589 (29.5)
Occupational injury or disease			
Occupational injury	799,521 (93.1)	638,965 (93.0)	160,556 (93.4)
Occupational disease	59,272 (6.9)	47,993 (7.0)	11,279 (6.6)
Duration from accident to approval			
<3 mo	556,315 (88.6)	446,339 (88.6)	109,976 (88.8)
3-6 mo	44,692 (7.1)	35,988 (7.1)	8,704 (7.0)
6-9 mo	12,979 (2.1)	10,404 (2.1)	2,575 (2.1)
9-12 mo	5,296 (0.8)	4,224 (0.8)	1,072 (0.9)
1-2 yr	6,199 (1.0)	5,010 (1.0)	1,189 (1.0)
2-3 yr	1,619 (0.3)	1,336 (0.3)	283 (0.2)
>3 yr	454 (0.1)	379 (0.1)	75 (0.1)
Duration from application to approval			
<3 mo	262,033 (98.4)	213,365 (98.4)	48,668 (98.7)
3-6 mo	3,570 (1.3)	2,973 (1.4)	597 (1.2)
6-9 mo	346 (0.1)	309 (0.1)	37 (0.1)
9-12 mo	158 (0.1)	151 (0.1)	7 (0.0)
1-2 yr	83 (0.0)	80 (0.0)	3 (0.0)
2-3 yr	4 (0.0)	4 (0.0)	0 (0.0)
>3 yr	3 (0.0)	3 (0.0)	0 (0.0)
Working year (yr)			
<5	730,508 (85.1)	579,348 (84.3)	151,160 (88.0)
5-10	61,589 (7.2)	47,986 (7.0)	13,603 (7.9)
10-15	29,221 (3.4)	24,677 (3.6)	4,544 (2.6)
15-20	18,017 (2.1)	16,451 (2.4)	1,566 (0.9)
20-25	10,512 (1.2)	9,910 (1.4)	602 (0.4)
25-30	5,540 (0.6)	5,315 (0.8)	225 (0.1)
>30	3,406 (0.4)	3,271 (0.5)	135 (0.1)
Industry			
Agriculture, forestry, and fishing	19,419 (2.3)	16,306 (2.4)	3,113 (1.8)
Mining	7,547 (0.9)	7,317 (1.1)	230 (0.1)
Manufacturing	304,214 (35.4)	260,916 (38.0)	43,298 (25.2)
Electricity, gas service	362 (0.0)	349 (0.1)	13 (0.0)
Water and waste services	430 (0.1)	398 (0.1)	32 (0.0)
Construction	195,559 (22.8)	188,585 (27.5)	6,974 (4.1)
Wholesale trade	53 (0.0)	43 (0.0)	10 (0.0)
Transport, postal, and warehousing	40,644 (4.7)	38,077 (5.5)	2,567 (1.5)
Retail trade and accommodation	62,559 (7.3)	31,412 (4.6)	31,147 (18.1)
Information media and telecommunications	5,832 (0.7)	4,906 (0.7)	926 (0.5)
Financial and insurance services	4,861 (0.6)	3,527 (0.5)	1,334 (0.8)
Real estate service	2,437 (0.3)	1,900 (0.3)	537 (0.3)
Professional, scientific, technical, administrative, and support services	6,913 (0.8)	5,222 (0.8)	1,691 (1.0)
Rental, hiring services	82,814 (9.6)	55,024 (8.0)	27,790 (16.2)
Public administration and safety	1,521 (0.2)	841 (0.1)	680 (0.4)
Education and training	16,132 (1.9)	3,138 (0.5)	12,994 (7.6)
Healthcare and social assistance	24,420 (2.8)	4,337 (0.6)	20,083 (11.7)
Arts, recreation, and other services	9,079 (1.1)	6,521 (0.9)	2,558 (1.5)
Associations, organizations, and other personal services	73,267 (8.5)	57,528 (8.4)	15,739 (9.2)
International and foreign organizations	482 (0.1)	369 (0.1)	113 (0.1)
Occupation			
Managers	55,053 (6.4)	48,788 (7.1)	6,265 (3.6)
Professionals and related workers	61,682 (7.2)	39,894 (5.8)	21,788 (12.7)
Clerks	61,284 (7.1)	45,254 (6.6)	16,030 (9.3)
Service workers	52,145 (6.1)	15,177 (2.2)	36,968 (21.5)
Sales workers	16,170 (1.9)	10,061 (1.5)	6,109 (3.6)
Skilled agricultural, forestry, and fishery workers	9,467 (1.1)	8,694 (1.3)	773 (0.4)
Craft and related trade workers	151,296 (17.6)	143,778 (20.9)	7,518 (4.4)
Plant, machine operators, and assemblers	121,506 (14.1)	111,579 (16.2)	9,927 (5.8)
Elementary workers	329,855 (38.4)	263,434 (38.3)	66,421 (38.7)
Approved disease			
Infectious and parasitic disease (A00-B99)	1,693 (0.2)	60 (3.5)	237 (14.0)
Neoplasm (C00-D48)	315 (0.0)	8 (2.5)	56 (17.8)
Immune disease, blood (D50-D89)	42 (0.0)	0 (0.0)	5 (11.9)
Endocrine, nutritional, and metabolic disease (E00-E90)	278 (0.0)	6 (2.2)	39 (14.0)
Mental disease (F00-F90)	470 (0.1)	7 (1.5)	55 (11.7)
Disease of the nervous system (G00-G99)	1,986 (0.2)	27 (1.4)	269 (13.5)
Eye disease (H00-H59)	3,473 (0.4)	42 (1.2)	416 (12.0)
Ear disease (H60-H95)	1,740 (0.2)	38 (2.2)	387 (22.2)
Cardiovascular disease (I00-I99)	6,555 (0.8)	391 (6.0)	1,385 (21.1)
Respiratory disease (J00-J99)	5,862 (0.7)	601 (10.3)	1,479 (25.2)
Digestive system (K00-K99)	1,131 (0.1)	21 (1.9)	120 (10.6)
Disease of the skin (L00-L99)	2,320 (0.3)	37 (1.6)	264 (11.4)
Musculoskeletal disease (M00-M99)	40,088 (4.7)	201 (0.5)	4,667 (11.6)
Urogenital disease (N00-N99)	198 (0.0)	3 (1.5)	33 (16.7)
Pregnancy, congenital malformation (O00-O99, P00-P99, Q00-Q99)	294 (0.0)	1 (0.3)	25 (8.5)
Injury, poisoning (S00-T98, V01-V98)	791,208 (92.1)	8,311 (1.1)	89,415 (11.3)
Other (R00-R99, U00-U99, Z00-Z99)	1,140 (0.1)	0 (0.0)	0 (0.0)
Severity of impairment			
No impairment	521,175 (60.7)	398,461 (58.0)	122,714 (71.4)
Mild	308,403 (35.9)	262,029 (38.1)	46,374 (27.0)
Moderate	25,097 (2.9)	22,687 (3.3)	2,410 (1.4)
Severe	4,117 (0.5)	3,780 (0.6)	337 (0.2)
CCI score (continuous), mean±SD (yr)^1^	0.4±0.7	0.4 ±0.7	0.4 ±0.7
CCI score (categorical)^1^			
0	563,999 (68.6)	449,326 (68.9)	114,673 (67.6)
≥1	257,992 (31.4)	203,121 (31.1)	54,871 (32.4)

Values are presented as number (%). SD, standard deviation; CCI, Charlson comorbidity index.

1 CCI score is a quantitative measure that predicts ten-year mortality for a patient who may have a range of comorbid conditions; Each condition is assigned a score based on the risk of dying associated with this condition; The total score is calculated by summing all of the applicable scores.

## DATA ACCESSIBILITY

The data used in this study are not freely available. Because of the Personal Information Protection Act and the NHIS database policy, only pre-authorized researchers can access cohort data. Proposals for possible collaboration for further data analysis should be sent to Professor Inah Kim (inahkim@hanyang.ac.kr).
